# Circ_0065149 Alleviates Oxidized Low-Density Lipoprotein-Induced Apoptosis and Inflammation in Atherosclerosis by Targeting miR-330-5p

**DOI:** 10.3389/fgene.2021.590633

**Published:** 2021-02-02

**Authors:** Dan Li, Wen Jin, Li Sun, Jiawei Wu, Hao Hu, Likun Ma

**Affiliations:** ^1^Division of Life Sciences and Medicine, The First Affiliated Hospital of USTC, University of Science and Technology of China, Hefei, China; ^2^Department of the 3rd Cardiovascular, Guangdong Second Provincial General Hospital, Guangzhou, China

**Keywords:** circular RNAs, circ_0065149, atherosclerosis, miR-330-5p, NF-kB signaling

## Abstract

**Background:**

Atherosclerosis is a risk factor for cardiovascular diseases. However, the roles of Circular RNAs (circRNAs) in atherosclerosis is unknown. Our study aimed to explore the effects of circ_0065149 in the pathogenesis of atherosclerosis.

**Methods:**

The expression of circ_0065149 ox-LDL-induced in human umbilical vein endothelial cells (HUVECs) was assessed by RT-PCR. Cell viability, lactate dehydrogenase leakage, apoptosis, invasion, and migration were assessed in HUVECs. Dual luciferase reporter system was carried out to determine the interaction between miR-330-5p and circ_0065149.

**Results:**

Our results showed that circ_0065149 was significantly lower in the ox-LDL-induced HUVECs. Overexpression of circ_0065149 promoted the cell viability and inhibited the apoptosis of ox-LDL-induced HUVECs. Overexpression of circ_0065149 also promoted the migration and invasion of ox-LDL-induced HUVECs. The expression of miR-330-5p was inhibited by overexpression of circ_0065149. Furthermore, circ_0065149 overexpression significantly inhibited the expressions of nuclear NF-κBp65 and suppressed the production of TNF-α, IL-6, and IL-1β in ox-LDL-induced HUVECs, which was rescued by the miR-330-5p mimic.

**Conclusion:**

These findings suggest that circ_0065149 plays an important role in the proliferation, apoptosis, and inflammatory response of HUVECs via targeting miR-330-5p.

## Introduction

Atherosclerosis is a global leading risk factor for ischemic cardiovascular, cerebrovascular, and peripheral arterial diseases (Herrington et al., 2016; Aufiero et al., 2019). The incidence of atherosclerosis-related diseases dropped in the developed world due to various therapeutic techniques. However, the risk of atherosclerosis remains high in both developed and developing countries. The key cellular mechanisms of atherosclerosis include the accumulation of vascular smooth muscle cells, endothelial damage, and lipid infiltration. Ox-LDL can induce dysfunction and apoptosis of endothelial cells by oxidative stress and thus forming atherosclerotic plaques (Pirillo et al., 2013; Pandey et al., 2014). Apoptosis of vascular endothelial cells is considered as the initial stage for AS pathogenesis.

Previous research has established that circRNAs are critical players in the development of cardiovascular diseases (Huang et al., 2019; Sun et al., 2020). Circ_0063517 regulated the angiogenesis of vascular endothelial cells in preeclampsia by targeting the miR-31-5p-ETBR axis (Li et al., 2020). Loss of circNifx promotes the myocardial function and prognosis in AMI mice (Huang et al., 2019). Knockdown of circCHFR inhibited the proliferation and migration of ox-LDL induced vascular smooth muscle cells (Yang et al., 2019). CircRNAs are also potential tools for the diagnosis and prediction of cardiovascular diseases. Another study showed that circTMEM56 and circDNAJC6 could act as biomarkers of disease severity in patients with hypertrophic cardiomyopathy (Sonnenschein et al., 2019). The roles of circRNAs in atherosclerosis are attracting more attention. Circ_0003204 is a key regulator in the proliferation and angiogenesis of HUVECs (Liu et al., 2020).

Hsa_circ_0065149 was downregulated in the oxLDL-induced human THP-1 macrophages by a previous study on profiling circRNAs (Wang et al., 2019). However, the biological effects of hsa_circ_0065149 on HUVECs remains to be validated. Here, our results showed that circ_0065149 was downregulated in ox-LDL-induced HUVECs. Overexpression of circ_0065149 inhibited the apoptosis and promoted the migration and invasion of ox-LDL-induced HUVECs. Our study provides novel insights into the mechanisms of circRNAs in atherosclerosis pathogenesis.

## Materials and Methods

### Cell Culture

Human umbilical vein endothelial cells (HUVECs) were bought from the Type Culture Collection of the Chinese Academy of Sciences (Shanghai, China) and were cultured in Dulbecco’s modified Eagle’s medium (DMEM, Hyclone) with 10% fetal bovine serum (FBS, Hyclone) at 37°C in incubator humidified with 5% CO_2_. HUVECs were treated with ox-LDL (100 μg/ml) to induce injury. A series of time points (0, 3, 6, 12, 24, and 48 h) were used to explore the dose-response relationship between the time of ox-LDL exposure and expression of circ_0065149. This study was approved by the Ethics Committee of The First Affiliated Hospital of USTC.

### Cell Transfection

HUVECs (5 × 10^6^ cells/well) were seeded into 6-well plates. To overexpress the circ_0065149, HUVECs were transfected with pcDNA3.1-circ_0065149 (OE circ_0065149) while the pcDNA3.1 empty vector served as a negative control. Plasmids were purchased from Genepharma (Shanghai, China). Transfection was performed using Lipofectamine 3000 (Invitrogen, CA, United States) strictly according to the manufacturer’s instructions.

### RT-PCR

Total RNA was extracted from HUVECs using the Trizol reagent (Thermo Fisher Scientific, Rockford, IL, United States) and quantified using the NANO 2000 ultraviolet spectrometer (Thermo Fisher Scientific, MA, United States). RNA was reverse-transcribed using the PrimeScript^TM^ RT Reagent Kit (Takara, Kyoto, Japan). PCR was performed with the SYBR Green PCR kit (TaKaRa) on the 7900HT Fast Real-Time PCR System (Applied Biosystems). GAPDH was used as the internal control.

### Cell Viability and Injury Analyses

The viability of HUVECs was determined using the Cell Counting Kit-8 (CCK-8) (Dojindo, Kumamoto, Japan). The absorbance at 450 nm was measured with a microplate reader Model 680 (Bio-Rad, California, United States). Cell injury was determined using the LDH Cytotoxicity Assay Kit (Beyotime, Shanghai, China). The absorbance at 490 nm was measured using a microplate reader.

### Caspase Activity Assay

Caspase activity was measured using the Caspase-3 Activity Assay Kit (Calbiochem, United States) according to instructions. Caspase activity was detected using the Cary Eclipse fluorescence spectrophotometer.

### Flow Cytometry

The apoptosis was determined using the Annexin V-FITC Apoptosis Detection Kit (Invitrogen, California, United States). HUVECs were stained with 5 μl Annexin V-FITC and 1 μl PI for 20 min in the dark. Apoptosis was analyzed using the FACScan flow cytometer (Becton Dickinson, NJ, United States).

### Wound-Healing Assay

HUVECs were seeded in six-well plates (5 × 10^4^ cells/well) and cultured until approximately 80% confluence. Wound gaps were created using a 200 μl pipette tip. Images were taken at 0 and 24 h after scratch using a microscope system (Olympus, Japan).

### Transwell Assay

24-well transwell chambers (Corning, Toledo, United States) were used for cell invasion. HUVECs were seeded at a density of 5 × 10^4^ cells into the upper chamber with a serum-free medium, and the bottom chambers were filled with medium containing 10% FBS. Following 48 h of incubation at 37°C, HUVECs on the underside were fixed with 4% formaldehyde solution and then stained with crystal violet. Ten random fields were counted using an optical microscope.

### Western Blot

The nuclear and cytoplasmic fractions were extracted from HUVECs with Nuclear and Cytoplasmic Protein Extraction Kit (Beyotime) following the manufacturer’s instructions. Briefly, cells were washed with iced PBS, collected in 200 μl of agent A and then left on ice for 15 min. Cells were then incubated in 10 μl of agent B and centrifuged at 12,000*g* for 10 min at 4°C. The resulting supernatants were retained as the cytoplasmic fraction. The pellets were resuspended in 50 μl of nuclear protein extraction buffer, votexed vigorously for 20 s, and left on ice for 3 min. The process was repeated six times. After centrifugation at 12,000*g* for 10 min at 4°C, the supernatant was collected as the nuclear fraction. The separated cytoplasmic and nuclear fractions were subjected to SDS-PAGE and transferred to PVDF membranes. The membranes were blocked in 5% non-fat milk and incubated with anti-NF-κB p65 and anti-IκBα (Abcam, United States) at 4°C overnight and were incubated with horseradish peroxidase-conjugated secondary antibody. Lamin B1 and α-Tubulin were used as loading controls. The bands were analyzed using the ImageJ software.

### ELISA

The supernatant of HUVECs was collected from each group. The levels of tumor necrosis factor (TNF-α), interleukin (IL)-6, and IL-1β were tested using ELISA test kits (Shanghai Tongwei Biological Technology Co., Ltd., Shanghai, China) according to instructions. The absorbance was measured at 450 nm using a microplate reader (Multiskan GO, Thermo Fisher Scientific, Inc.).

### Luciferase Activity Assay

Starbase 2.0 and TargetScan were used to predict the binding sites between circ_0065149 and miR-330-5p. The wild and mutant circ_0065149 were synthesized by Shanghai GenePharma (Shanghai, China) and cloned into the Renila luciferase reporter vectors (Promega, Madison, United States). The NRK-52E cells were co-transfected with miR-330-5p mimics or mimic-NC. The luciferase activity was detected using a dual-luciferase reporter assay system (Promega) with Renilla luciferase as the internal control.

### Statistical Analysis

Statistical analyses were performed with SPSS (V22.0, IBM, United States). Data were shown as means and standard deviations. The differences between groups were compared using the *t*-test. A *p* < 0.05 was considered statistically significant.

## Results

### Overexpression of Circ_0065149 Promoted the Viability of ox-LDL-Induced HUVECs

**FIGURE 1:**
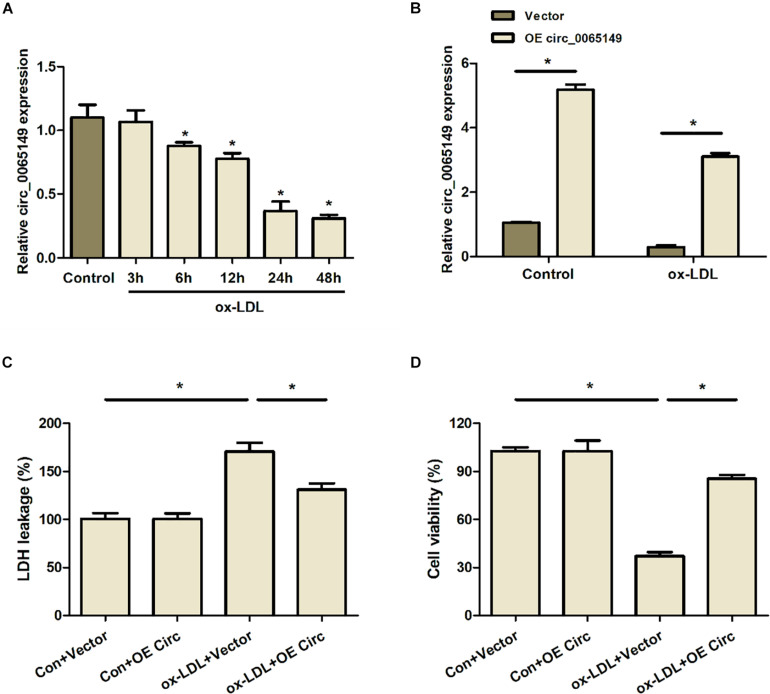
Overexpression of circ_0065149 promoted the viability of HUVECs treated with ox-LDL. **(A)** Expression of circ_0065149 in HUVECs was measured by qRT-PCR. *P < 0.05 vs. the Control group. **(B)** Expression of circ_0065149 in HUVECs transfected with Vector or OE circ_0065149. *P < 0.05 vs. the Vector group. **(C)** LDH leakage was detected using an LDH Cytotoxicity Assay Kit. *P < 0.05, ox-LDL + Vector vs. the Con-Vector group; ox-LDL + OE Circ vs. the ox-LDL + Vector group. **(D)** Cell viability was measured by a CCK-8 assay. *P < 0.05, ox-LDL + Vector vs. the Con-Vector group; ox-LDL + OE Circ vs. the ox-LDL + Vector group. Data are shown as means ± *SD*.

We first measured the expression of circ_0065149 in HUVECs after treatment with ox-LDL. We found that the expression of circ_0065149 was significantly downregulated in HUVECs treated with ox-LDL than those without and continued to decrease within 40 h (Figure 1A). Therefore, 48 h of ox-LDL treatment was used in subsequent experiments. As shown in Figure 1B, the relative expression of circ_0065149 was effective ever-expressed by OE circ_0065149 in both groups. Treatment of ox-LDL boosted the leakage of LDH in HUVECs, which was restored by overexpression of circ_0065149 (Figure 1C). Ox-LDL exposure also suppressed the cell viability of HUVECs, which was reversed by circ_0065149 overexpression (Figure 1D). These data suggested that circ_0065149 promoted the viability of ox-LDL-induced HUVECs.

### Overexpression of Circ_0065149 Inhibited the Apoptosis of ox-LDL-Induced HUVECs

**FIGURE 2:**
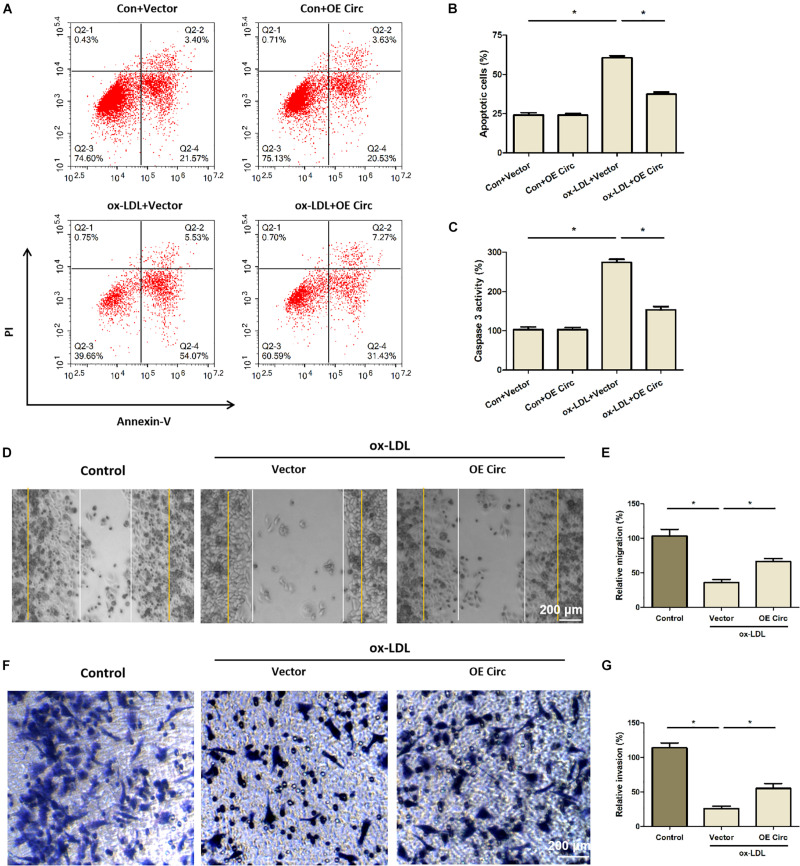
Overexpression of circ_0065149 inhibited the apoptosis and promoted the migration and invasion of HUVECs treated with ox-LDL. **(A,B)** Apoptosis of HUVECs was measured by flow cytometry. *P < 0.05, ox-LDL + Vector vs. the Con-Vector group; ox-LDL + OE Circ vs. the ox-LDL + Vector group. **(C)** Caspase 3 activity assay. *P < 0.05, ox-LDL + Vector vs. the Con-Vector group; ox-LDL + OE Circ vs. the ox-LDL + Vector group. **(D,E)** Migration was detected by wound healing assay at 0 (yellow line) and 24 h (white line). Bar indicated 200 μm. *P < 0.05, Vector vs. the Control group; ox-LDL + OE Circ vs. the ox-LDL Vector group. **(F,G)** Invasion was detected by Transwell assay. *P < 0.05, Vector vs. the Control group; ox-LDL + OE Circ vs. the ox-LDL Vector group. Data are shown as means ± *SD*.

The results of flow cytometry revealed that ox-LDL-stimulation raised the apoptosis of HUVECs, which was restored partially by the overexpression of circ_0065149 (Figures 2A,B). Also, ox-LDL treatment increased the caspase 3 activity in HUVECs, whereas this phenomenon was alleviated by the overexpression of circ_0065149 (Figure 2C). Interestingly, circ_0065149 overexpression had little effect on the cell viability and apoptosis of HUVECs without ox-LDL treatment. These data suggest that overexpression of circ_0065149 inhibited the apoptosis of ox-LDL-induced HUVECs.

### Overexpression of Circ_0065149 Promoted the Migration and Invasion of HUVECs

Results of the wound closure assay showed that the migration capability of ox-LDL-induced HUVECs was significantly decreased compared to the control group, whereas this change was partially restored by overexpression of circ_0065149 (Figures 2D,E). Similarly, the invasion capability was significantly decreased in ox-LDL-induced HUVECs and was partially restored by overexpression of circ_0065149 (Figures 2F,G).

### Overexpression of Circ_0065149 Inhibited the NF-κB Pathway and the Production of Proinflammatory Cytokines

**FIGURE 3:**
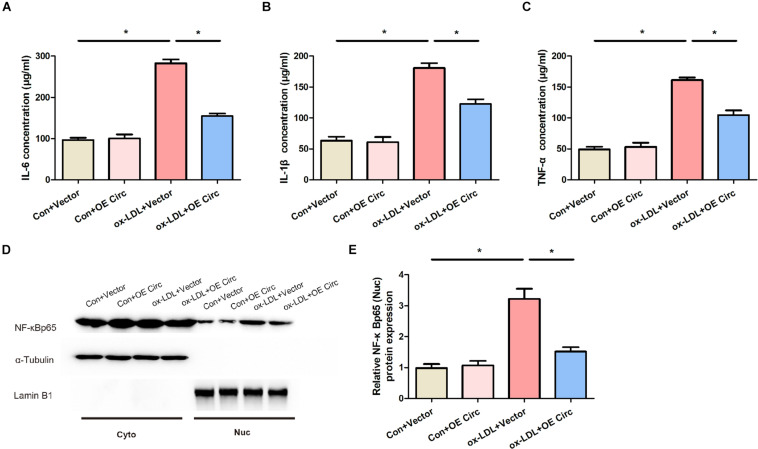
Overexpression of circ_0065149 decreased the expressions of IL-6 **(A)**, IL-1β **(B)**, TNF-α **(C)**, and NF-κBp65 **(D,E)** in ox-LDL-induced HUVECs. The expression of NF-κBp65 was assessed in the nuclear and cytoplasmic fractions, respectively. Data are presented as means ± *SD*. *P < 0.05, Vector vs. the Control group; ox-LDL + OE Circ vs. the ox-LDL Vector group.

To further study the mechanism of hsa_circ_0065149 in apoptosis and inflammation, we measured the expression of NF-κB, TNF-α, IL-6, and IL-1β using Western blot and ELISA (Figure 3). We found that the expressions of TNF-α, IL-6, and IL-1β were markedly increased by ox-LDL treatment and were partially reversed by overexpression of circ_0065149 (Figures 3A–C). Stimulation with ox-LDL resulted in a significant increase in the expression of nuclear NF-κBp65, which was reversed by the overexpression of circ_0065149 (Figures 3D,E). We also found that there was no significant difference in IκBα among the groups (Supplementary File). These data suggest that circ_0065149 overexpression inhibited the NF-κBp65 nuclear translocation and the production of proinflammatory cytokines in ox-LDL-induced HUVECs.

### Circ_0065149 Regulated the Levels of NF-κB and Proinflammatory Cytokines via Targeting miR-330-5p

**FIGURE 4:**
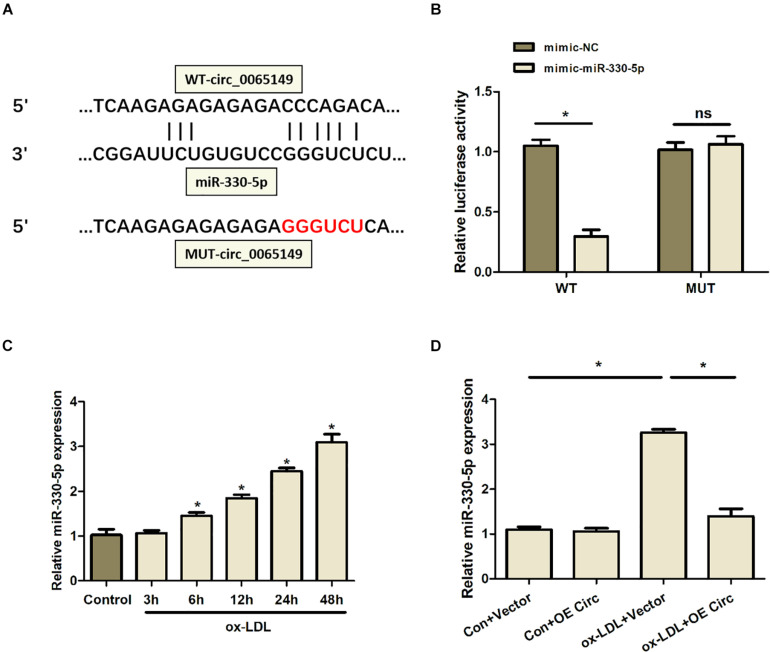
Overexpression of circ_0065149 decreased the expression of miR-330-5p in ox-LDL-induced HUVECs. **(A)** Predicted binding sites between circ_0065149 and miR-330-5p. **(B)** Luciferase activity of circ_0065149 in ox-LDL-induced HUVECs transfected with miRNA mimics. *P < 0.05, mimic-miR330-5p vs. the mimic-NC group; ox-LDL + OE Circ vs. the ox-LDL Vector group. **(C)** Expression of miR-330-5p in ox-LDL-induced HUVECs over time. *P < 0.05 vs. the Control group. **(D)** Expression of miR-330-5p in HUVECs treated with different conditions. *P < 0.05, Vector vs. the Control group; ox-LDL + OE Circ vs. the ox-LDL Vector group. Data are shown as means ± *SD*.

To investigate the mechanisms of the effects of circ_0065149, we predicted the targets of circ_0065149 by bioinformatics analysis. The predicted binding sites between circ_0065149 and miR-330-5p were shown in Figure 4A. Luciferase reporter assays showed that the luciferase activities of circ_0065149 wild type were significantly decreased when transfected with miR-330-5p mimics compared with the control reporter (Figure 4B). The expression of miR-330 was significantly increased with time in ox-LDL HUVECs (Figure 4C). We also found that the expression of miR-330 was inhibited by the overexpression of circ_0065149 (Figure 4D). These results implied that miR-330-5p served as a target for circ_0065149.

**FIGURE 5:**
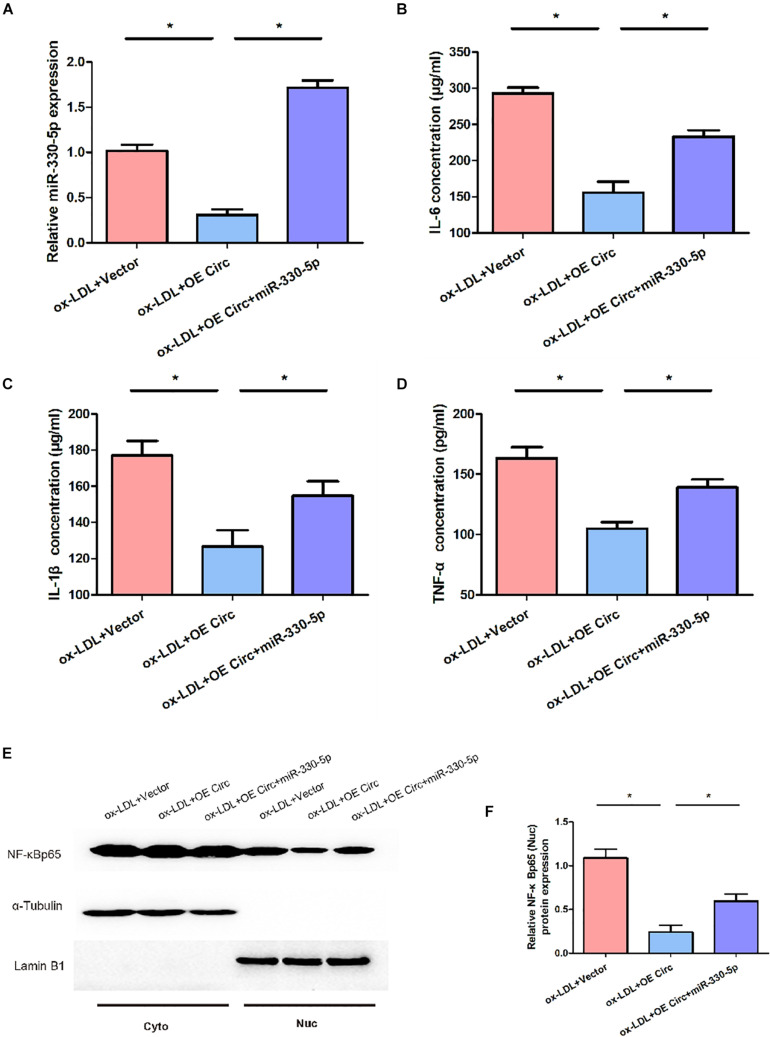
Effect of miR-330-5p on the levels of NF-κB and proinflammatory cytokines in ox-LDL-induced HUVECs. **(A)** Expression of miR-330-5p in ox-LDL-induced HUVECs. MiR-330-5p mimic increased the expressions of IL-6 **(B)**, IL-1β **(C)**, TNF-α **(D)** in ox-LDL-induced HUVECs. **(E,F)** MiR-330-5p mimic restored the nuclear expression of NF-κBp65 in ox-LDL-induced HUVECs. The expression of NF-κBp65 was assessed in the nuclear and cytoplasmic fractions. Data are shown as means ± *SD*. *P < 0.05, ox-LDL + OE Circ vs. the ox-LDL Vector group; ox-LDL + OE Circ + miR330-5p vs. the ox-LDL Vector group.

The miR-330-5p mimic successfully restrained the effects of circ_0065149 on the expression of miR-330-5p (Figure 5A). The miR-330-5p mimic reduced the circ_0065149-mediated suppression on the levels of TNF-α, IL-6, and IL-1βin ox-LDL-induced HUVECs (Figures 5A–D). Besides, the expression of nuclear NF-κBp65 was suppressed by circ_0065149 overexpression, which was reversed by the miR-330-5p mimic. These results confirmed that circ_0065149 functioned by sponging miR-330-5p in ox-LDL-induced HUVECs.

## Discussion

Recent evidence suggests that circRNAs play a crucial role in the development of a variety of heart diseases (Zhang et al., 2018; Altesha et al., 2019; Tang et al., 2020). Deep RNA sequencing of cardiac tissue revealed differential expression of circRNAs in the heart (Tan et al., 2017). It has been shown that over-expression of circFndc3b improved heart function by binding to the RNA binding protein FUS (Garikipati et al., 2019). The level of hsa_circ_0124644 in peripheral blood was shown to be a potential biomarker for diagnosing coronary artery disease (Zhao et al., 2017). Our study focused on and investigated the role of circ_0065149 on ox-LDL-induced HUVECs. Using RT-PCR, we found that circ_0065149 was notably downregulated in HUVECs treated with ox-LDL, which was similar to a previous study that reported downregulation of circ_0065149 in the ox-LDL-induced macrophages (Wang et al., 2019). Hence, it could conceivably be hypothesized that circ_0065149 may be involved in the pathogenesis of atherosclerosis.

The present study found that overexpression of circ_0065149 boosted the proliferation, migration, and invasion and inhibited the apoptosis of ox-LDL-induced HUVECs. Vascular endothelial cells are the innermost layer of blood vessels. Therefore, dysfunction of endothelial cells is considered an initial step in the pathogenesis of atherosclerosis. When endothelial cells are exposed to various pathogenic threats, such as ox-LDL, abnormal proliferation, migration, and angiogenesis occur, resulting in the destruction of endothelial integrity, thus aggravating lipid deposition and fibrous cap rupture (Badimon and Vilahur, 2014). CircRNAs have been reported to regulate the apoptosis and survival of cells in heart diseases. Another study has reported that over-expression of circFndc3b reduces the apoptosis of cardiomyocytes and endothelial cells, thus improving myocardial function (Garikipati et al., 2019). Circular hsa_circ_0003204 are involved in the proliferation, migration, invasion, and apoptosis of ox-LDL-induced HUVECs (Liu et al., 2020). The silencing of cZNF292 suppressed the proliferation and tube formation capability of HUVECs *in vitro* (Boeckel et al., 2015). A study has reported that circ_0003204 inhibited the proliferation, migration, and tube formation of HAECs treated with ox-LDL (Zhang et al., 2020). Additionally, testing the circ_0003204 in plasma extracellular vesicles may be a potential tool for predicting the diagnosis and prognosis of cerebral atherosclerosis (Zhang et al., 2020). The data of the present study infer that overexpression of circ_0065149 has a protective effect on vascular endothelial cells, thus preventing atherosclerosis.

In response to these findings, we subsequently investigated the mechanisms by which circ_0065149 regulates the activities of HUVECs. The NF-κB signaling pathway regulates inflammatory responses and is related to atherosclerosis (de Winther et al., 2005). The NF-κB is increased in all major cell types in the atherosclerotic plaques (de Winther et al., 2005). Another *in vivo* study revealed that endothelial NF-κB signaling modulates the expression of proinflammatory genes in the arterial wall and promotes the development of atherosclerosis (Gareus et al., 2008). Inhibition of endothelial intrinsic NF-κB signaling mitigates atherosclerosis caused by chronic intermittent hypoxia (Song et al., 2018). In our study, circ_0065149 overexpression inhibited the nuclear translocation of NF-κBp65 and the expressions of TNF-α, IL-6, and IL-1β in ox-LDL-induced HUVECs. Using luciferase activity assays, we found the binding sites between circ_0065149 and miR-330-5p. Furthermore, the miR-330-5p mimic decreased the circ_0065149-mediated suppression of apoptosis and inflammation. These results confirmed that circ_0065149 regulated the NF-κB signaling pathway and the production of proinflammatory cytokines by targeting miR-330-5p.

Several limitations should be noted about the present study. Firstly, we only used an oxLDL-mediated HUVECs damage model. We did not evaluate whether circ_0065149 overexpression can alleviate HUVECs damage caused by other factors. Secondly, we explored the protective effects of circ_0065149 *in vitro*. Animal models should be considered in further research to confirm our findings.

In summary, our study showed that the expression of circ_0065149 decreased ox-LDL-induced HUVECs. Overexpression of circ_0065149 promoted the proliferation, migration, invasion, and inhibited the apoptosis of endothelial cells. Our findings reveal that that circ_0060745 alleviated the apoptosis and inflammation induced by ox-LDL via sponging miR-330-5p. These findings provide a novel target for treating endothelial cell damage in atherosclerosis.

## Data Availability Statement

The original contributions presented in the study are included in the article/Supplementary Material, further inquiries can be directed to the corresponding author/s.

## Author Contributions

DL, WJ, and LM conceived, designed the study, and revised the manuscript. DL, WJ, LS, JW, and HH performed the experiments and analyzed the data. All authors approved the final version of the manuscript.

## Conflict of Interest

The authors declare that the research was conducted in the absence of any commercial or financial relationships that could be construed as a potential conflict of interest.
