# Supplementary material for: Circ_0065149 Alleviates Oxidized Low-Density Lipoprotein-Induced Apoptosis and Inflammation in Atherosclerosis by Targeting miR-330-5p
Source: Front Genet. 2021 Feb 2;12:590633. doi: 10.3389/fgene.2021.590633 (PMC7884639; doi:10.3389/fgene.2021.590633)
Supplement: Supplementary file 1 [file Data_Sheet_1.PDF]

Fig 3. NF-κBp65

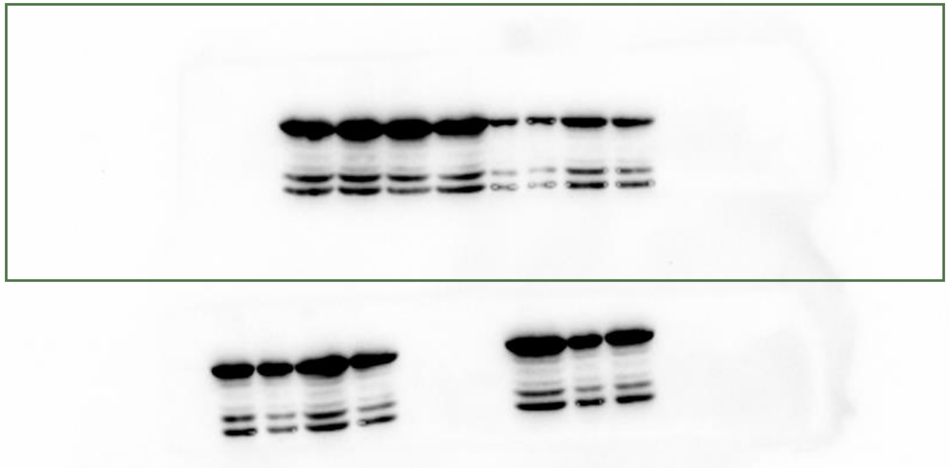

Fig 3. α-Tubulin

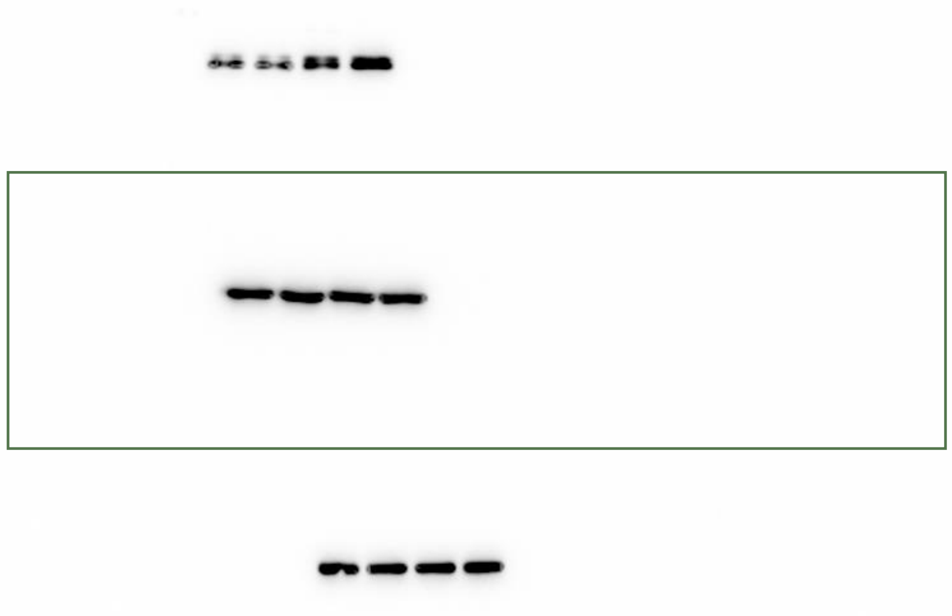

Fig 3. Lamin B1

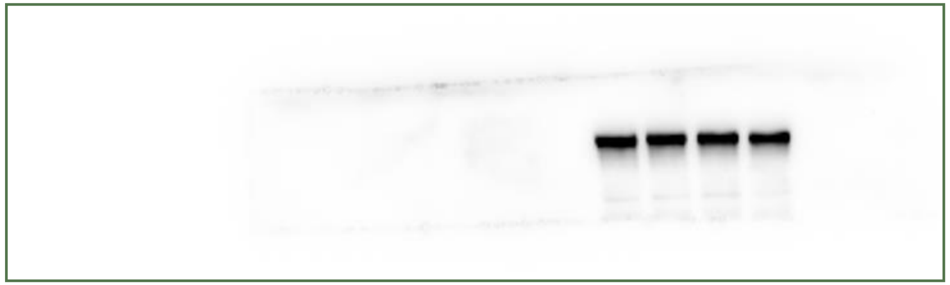

NF-κBp65

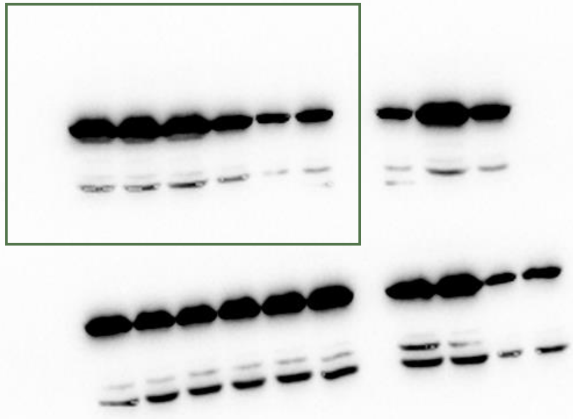

α-Tubulin

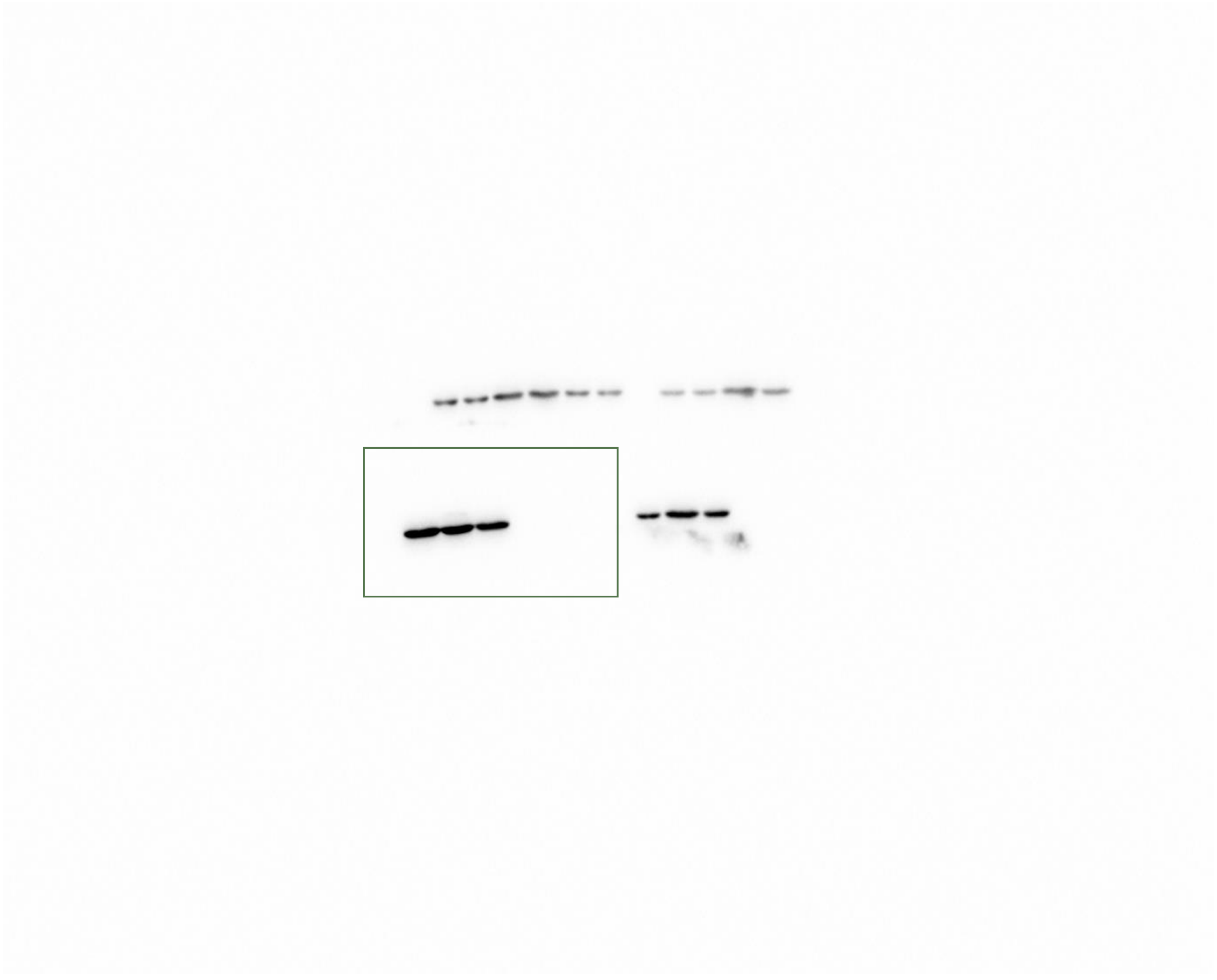

Lamin B1

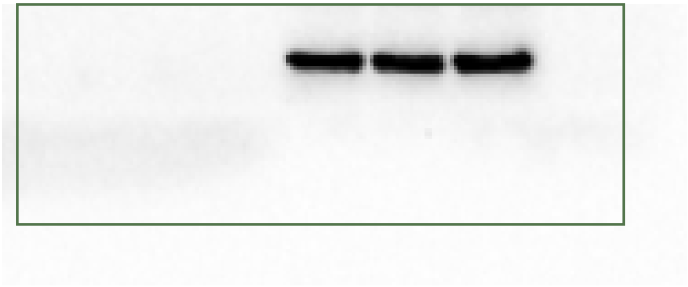

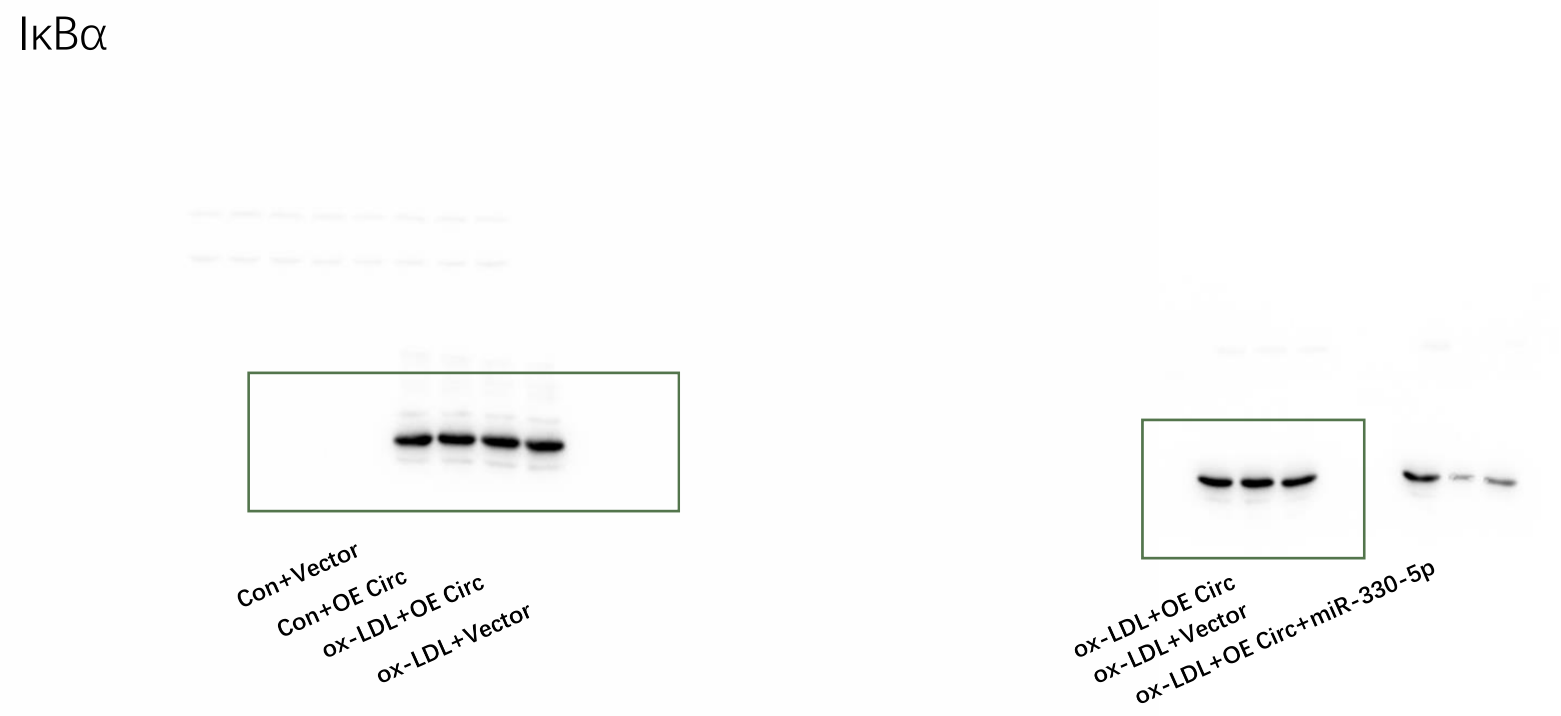
IκBα
Con+Vector
Con+OE Circ
ox-LDL+OE Circ
ox-LDL+Vector
ox-LDL+OE Circ
ox-LDL+Vector
ox-LDL+OE Circ+miR-330-5p
